# Supplementary material for: Proteomic Study of Low-Birth-Weight Nephropathy in Rats
Source: Int J Mol Sci. 2021 Sep 24;22(19):10294. doi: 10.3390/ijms221910294 (PMC8508940; doi:10.3390/ijms221910294)
Supplement: Supplementary file 1 [file ijms-22-10294-s001.zip › SI .pdf]

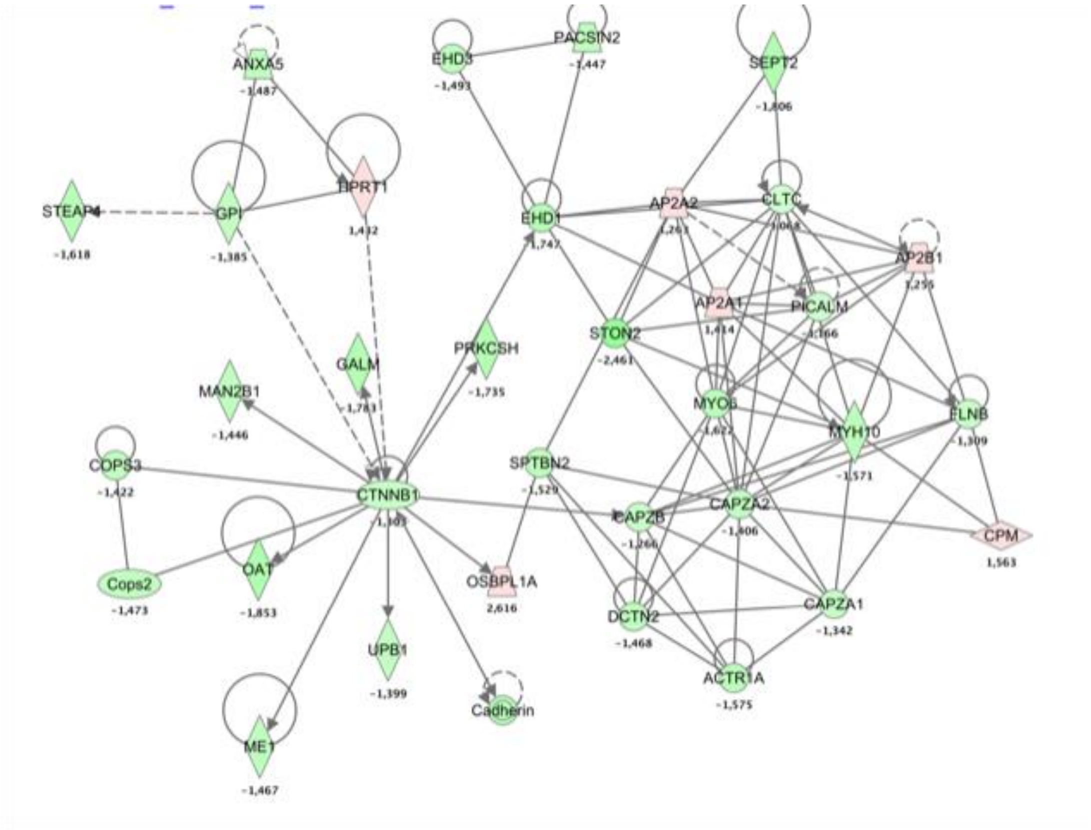

Figure S1. Network of proteins involved in the regulation of cell architecture altered in the LBW kidney. Green = protein repressed and pink = protein upregulated in the LBW proteomics data.

Table S1. Differential proteomics quantitative data. (A) The normalized content for each protein was determined on N=7 animals per group. ANOVA was performed to calculate the *p value* of the difference in protein content for each protein detected in the analysis between the LBW and NBW groups. The fold change (LBW/NBW) is given in the table. (B) Multiple comparison analysis with Benjamini and Hochberg correction was performed on the raw proteomic dataset to calculate the adjusted p value (q value).

Table S2. Pathway and regulator IPA analysis of the LBW proteomic dataset. Results of the ‘core analysis’ (A) ‘pathways’ and (B) ‘regulators’ analysis of the LBW kidney proteome obtained using IPA (Qiagen). For (A) and (B) the background gene-set for “Core Analysis” was set to “Ingenuity Knowledgebase”. For (C) the background gene-set for “Core Analysis” was set to “authors dataset”.
